# Comparison of Instrumented Mouthguard Post-Processing Methods

**DOI:** 10.1007/s10439-025-03687-1

**Published:** 2025-02-22

**Authors:** Ryan Gellner, Mark T. Begonia, Matthew Wood, Lewis Rockwell, Taylor Geiman, Caitlyn Jung, Blake Gellner, Allison MacMartin, Sophia Manlapit, Steve Rowson

**Affiliations:** 1 Virginia Tech (Biomedical Engineering and Mechanics), Blacksburg, VA USA; 2 Carnegie Mellon (Mechanical Engineering), Pittsburgh, PA USA; 3 Wayne State University (Biomedical Engineering), Detroit, MI USA

**Keywords:** Instrumented mouthguard, Post-processing, Labeling, Head impact, Salvage, Artifact

## Abstract

Instrumented head acceleration measurement devices are commonly used in research studies to determine head acceleration exposure in certain populations. Instrumented mouthguards pair directly to the user’s teeth and offer six-degree-of-freedom measurements. Though many studies have recently used these devices, post-processing techniques vary by study. Other studies have attempted to label impact quality or coupling status, also with varying methods. This study sought to compare the effect of post-processing and labeling methods on reported exposure distribution characteristics in instrumented mouthguard data from ice hockey players. We collected data from 18 female adolescent ice hockey players on two teams for an entire season. We then post-processed the measured signals using five different techniques: (1) the instrumented mouthguard manufacturer’s data output, (2) a 500 Hz linear acceleration filter and a 300 Hz angular velocity filter, (3) HEADSport, (4) a 100 Hz linear acceleration filter and a 175 Hz angular velocity filter, and (5) a salvaging process to detect and remove decoupling based on signal frequency content. The post-processing techniques affected the reported exposure distributions by changing the mean, median, and 95th percentile values of peak linear and angular kinematics. We also compared labeling techniques by measuring agreement and inter-rater reliability between three labeling techniques: the instrumented mouthguard manufacturer’s label, Luke et al.’s coupling label, and our classification learner that detects and labels decoupling. We found that the labeling techniques had low agreement about which acceleration events were the best to keep. Labeling technique also influenced the reported distributions’ descriptive statistics. Post-processing and event labeling are crucial components of head acceleration event exposure studies. Methods should be described by researchers, and standardization should be sought to allow for better cross-study comparison. Published and publicly available techniques can help move the field toward this ideal. Researchers should be aware of the potential effect post-processing can have on a population’s final reported exposure metrics.

## Introduction

Mild traumatic brain injuries, better known as concussions, have been studied in biomedical engineering settings for decades. Though these concussive injuries have always had the emphasis [1–6], recent studies have begun to focus on sub-clinical, or sub-concussive, head acceleration events (HAEs) as a potential contributor to long-term clinical sequelae [7–9]. As a result, biomedical researchers have begun to report head acceleration events and non-impact acceleration events as these acceleration events are experienced by volunteer athletes using a variety of head kinematic sensors. Volunteer athletes offer an ideal opportunity to study head impacts in living people because they are subject to head impacts while playing their sports simply by means of legal contact occurring during practice or play.

Head kinematic sensors began with rudimentary, wired sensors [10, 11] and evolved to wireless, on-field sensors for sport and military applications. Today’s instrumented mouthpieces feature highly relevant six-degree-of-freedom motion data [12–15]. In addition, battery life and usability have improved so that volunteer athletes can wear the devices for hours, and researchers can download the data easily, often to a mobile app.

Various commercial and non-commercial devices have been used in recent research, with differing levels of reliability and accuracy [15–17]. Many sensors have been tested in a laboratory setting and on the field with volunteer athletes, with promising levels of accuracy in reported kinematics. However, no standard or agreement exists regarding appropriate post-processing methods for these devices, which may influence their overall accuracy [18, 19].

Commercial instrumented mouthguards (iMGs) are post-processed using proprietary filtering methods that are often a black box to the researcher using the data [15, 20]. Recent literature suggests that these filtering methods may not be adequate to capture accurate signal content from head kinematics [18, 21]. Specialized noise artifact-removal techniques have been suggested to filter iMG signals based on the specific frequency content of the signal [19, 22]. One of these techniques, HEADSport, has been made publicly available on GitHub.

Additionally, iMG signal quality labeling has begun to be reported for field data [20, 23]. Labeling signal quality or reliability is important because it allows researchers and reviewers to understand the relative weight to place on the data from a given impact. Commercial iMG companies have their own “quality” scores for acceleration events, the details of which are often hidden from researchers. Luke et al. recently proposed a method for labeling signal quality based on an infrared proximity sensor reading taken just before and after an impact [23]. Machine learning approaches also show promise for the ability to classify signals with artifacts [24].

Our study aimed to quantify differences in head impact kinematics in a collection of female ice hockey field data from commonly used post-processing techniques for iMGs. Additionally, we sought to measure the effect of labeling data quality on reported distributions. This study used female ice hockey field data as an exemplar dataset, which is meant to demonstrate the potential effects in field data distributions from other sports and populations.

## Methods

### Field Data Collection

We recruited 18 female ice hockey players (age: 16.1 ± 0.90 yr, height: 166 ± 7.87 cm, weight: 65.2 ± 9.60 kg) for participation in this study during the 2021-2022 season. Seven players came from an elite AAA national-bound team (Elite), and the remaining 11 from a high school team (High School) participating in the Michigan Girls High School Hockey League. The Michigan Amateur Hockey Association (MAHA) allows players in the state to be on only one team at a time, so athletes in this study were not playing for any other teams during the study period. Data were collected across 127 practices and games. Recruitment took place on a voluntary basis after informed consent at a team meeting. Volunteer athletes under the age of 18 gave assent and their parent or guardians gave consent. Volunteer athletes aged 18 or older gave their own consent. All data were collected in accordance with the Virginia Tech Institutional Review Board’s (VT IRB) rules and ethical standards under VT IRB number 21-500.

**Fig. 1.**
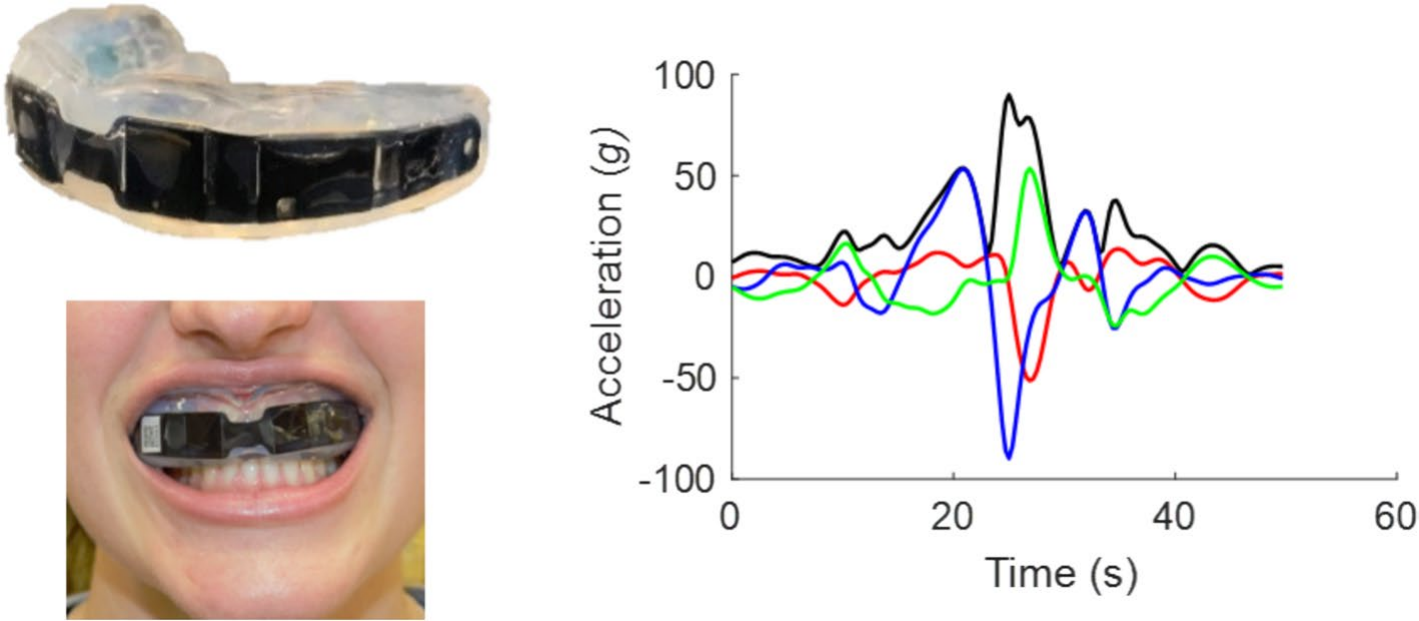
Instrumented mouthguard and example signal generated during an accelerative event.

Volunteer athletes were assigned a Prevent Biometrics (Prevent Biometrics, Edina, MN; firmware version 2.0.14) boil-and-bite style iMG for the season (Figure 1). This style and brand of iMG has a 0.97 CCC for peak linear acceleration and 0.98 CCC for peak angular acceleration when compared to laboratory reference values [16]. Additionally, this device has a positive predictive value (PPV) of 81.6% in identification of field impacts once events recorded outside of session times are removed [16]. Fitting took place according to the manufacturer’s instructions, which consisted of boiling the device for 20 s, having the participant bite the iMG for 30 s, and then having them create suction on the iMG for another 30 s. We deemed the fit acceptable if the iMG stayed on the teeth with an open mouth after this fitting process, as recommended by the manufacturer.

These iMGs have a three axis linear accelerometer (200 g) and a three-axis gyroscope (35 rad/s) [25], all measuring at a 3200 Hz sampling rate. The iMGs triggered when the linear acceleration on any single axis (in the iMG’s local coordinate system) exceeded 8 g. Upon triggering, the iMGs recorded 10 ms of pre-trigger and 40 ms of post-trigger data. Proximity data are recorded via an infrared proximity sensor to determine how close the inner surface of the iMG is to another surface (e.g., the teeth) during use. The proximity sensor uses a variable sampling rate (1–10 Hz) that is elevated when a pre-set proximity unit threshold is crossed and is highest during a triggered event. Data were downloaded live during the session via Bluetooth to a local iOS device running the Prevent Biometrics application. We collected the iMGs after each session to ensure all data were downloaded and the batteries were fully charged before the next session. We also inspected all iMGs to ensure they were functioning and replaced any non-functioning devices before the next session.

The iMGs recorded 18,971 acceleration events in 127 sessions (93 Elite, 34 High School). Using our records of session timing, we removed acceleration events outside of session times: before and after practices or games and between periods during games. We considered acceleration events non-trivial if the peak linear acceleration was greater than 5 g and the peak angular acceleration was greater than 400 rad/s^2^. [18, 26] Finally, any acceleration events not above 10 g peak resultant linear acceleration were removed because common tasks have been shown to record accelerations of this magnitude [27]. Our final dataset consisted of 6232 acceleration events, 4390 from the Elite team (70%) and 1842 from the High School team (30%) (Table 1).

**Table 1.** Acceleration events by team after data parsing

Team	Players	Acceleration events measured	Events remaining after removing outside session times	Events remaining after trivial events removed [26]	Final events remaining* after removing events < 10 g [27]
Elite	8	11218	6345	4696	4390
High School	11	5193	3209	1913	1842

*Final number of events: 6232, which is the sum of the events remaining from both teams after selection process.

### Post-Processing Technique Comparison

**Fig. 2.**
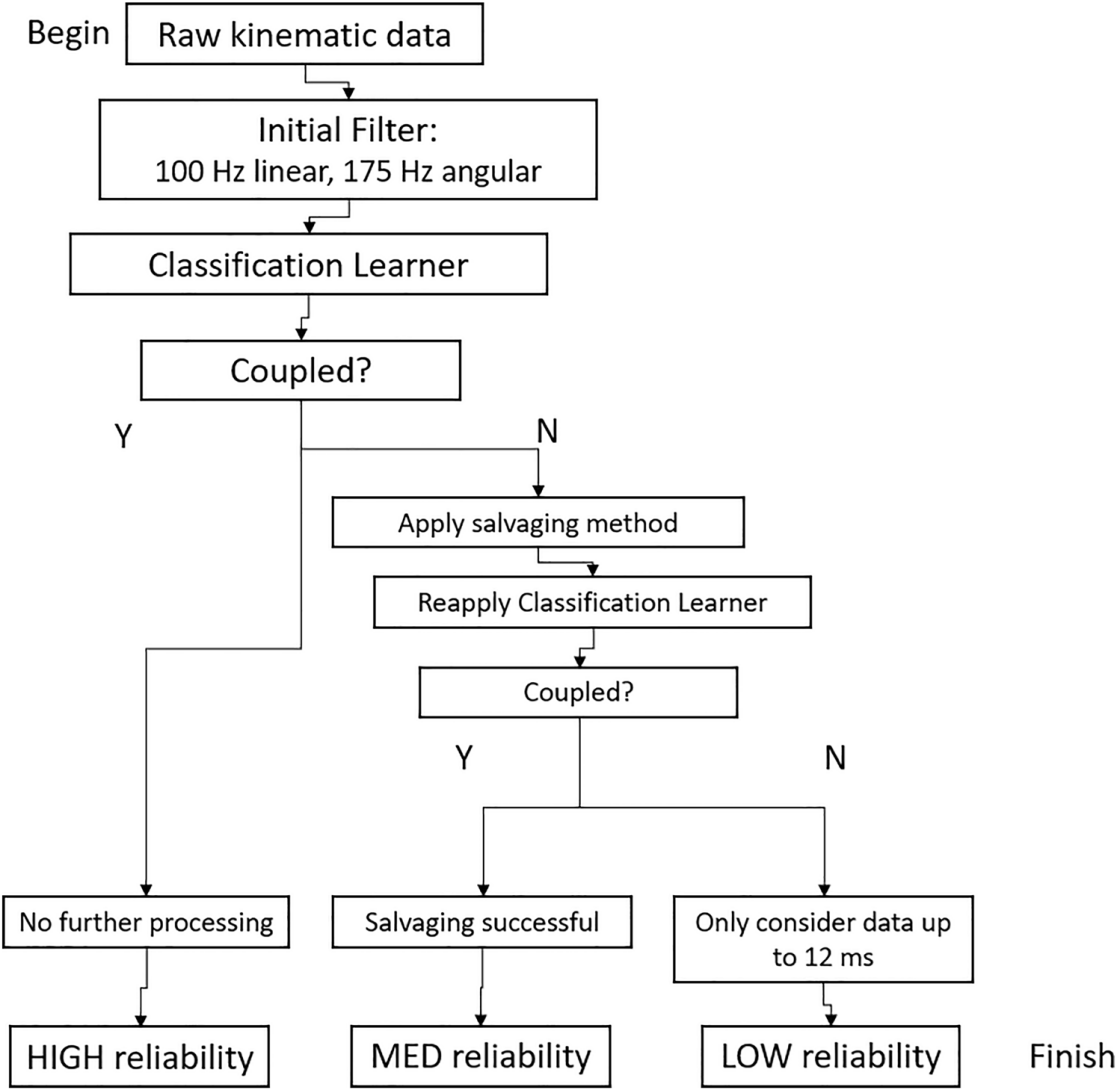
*Flowchart for our Salvaging method for measured head acceleration events. The Salvaging method consisted of wavelet denoising when decoupling was detected.* [24]

Raw iMG data were sent to our research team for analysis in addition to the manufacturer’s processing of each head acceleration event. No proprietary post-processing algorithms were performed on the raw data traces before we applied the described techniques. The manufacturer’s technique was taken from the processed data which we received in addition to the raw data for each event. Raw iMG kinematic traces that met the inclusion criteria were post-processed using five different techniques (Table 2): (1) the iMG manufacturer’s proprietary technique (manufacturer) was left “as is”; (2) we applied a 500 Hz linear and 300 Hz angular cutoff frequency combination [21] in a phaseless 4th-order Butterworth filter [28, 29]; (3) we used HEADSport [19]; (4) we applied a 100 Hz linear and 175 Hz angular cutoff frequency combination [18]; and (5) we used our Salvaging method that was developed using a classification learner algorithm [24] (Figure 2). We chose the 100 Hz linear and 175 Hz angular cutoff frequency combination from Gellner et al. because the acceleration events in our dataset were from ice hockey players wearing helmets; therefore, we assumed the optimal padded filter would apply because ice hockey impact durations are of similar duration to those in the padded category from that previous study [18, 30]. Angular acceleration was calculated from angular velocity via five-point stencil method. [31]

**Table 2.** Five post-processing techniques were compared to determine their effect on IMG acceleration event distributions

Technique	Linear cutoff frequency	Angular cutoff frequency	Varies by impact	Quality label
Manufacturer	200, 100, or 50 Hz	200, 100, or 50 Hz	Yes	Yes
Wu et al. [21]	500 Hz	300 Hz	No	No
HEADSport	30–1500 Hz	30–1500 Hz	Yes	No
Gellner et al. [29]	100 Hz	175 Hz	No	No
Salvaging	100 Hz + Wavelet denoising*	175 Hz + Wavelet denoising*	Yes	Yes

*Wavelet denoising performed if classification is decoupled—see Fig. 2

We compared post-processing techniques by determining their mean, standard deviation, median, and 95th percentile peak kinematic values. We also compared techniques via ordinary least products (OLP) regression of peak values, because each technique was assumed to have error [32]. We chose to run a Kruskal–Wallis rank-sum test for differences in distributions, as the distributions were skewed. We then applied a Bonferroni-corrected pairwise Wilcoxon test for pairwise differences.

We used the angular velocity change index (AVCI, also known as rotational velocity change index, RVCI [33]) rather than angular velocity because angular velocity signals were not used to trigger the iMG’s recording. This means an impact could be recorded when the head’s angular velocity is non-zero, biasing the peak angular velocity [34]. AVCI maximizes the change in angular velocity over an interval of up to 10 ms, preventing bias from the lack of angular velocity triggering.

### Impact Labeling Comparison

We also explored how impact labeling affected reported HAE distributions. Three labeling techniques were compared: (1) the manufacturer’s proprietary Quality label, (2) Luke et al.’s coupling label [23], and (3) our Salvaging method’s reliability flag. The iMG manufacturer labels acceleration events as having a Quality score of 0 (best), 1, or 2 (worst). Acceleration events with a score of 0 have their linear and angular signals filtered with a 200 Hz cutoff frequency, 1-score acceleration events use a 100 Hz filter cutoff frequency, and 2-score signals use a 50 Hz filter cutoff frequency [20]. Though the exact methods are unknown, the manufacturer may use proximity measurements and signal features to determine signal quality [24].

Luke et al. suggested using an individualized proximity threshold for each iMG to determine pre-impact and post-impact coupling status. This enabled him to label acceleration events into one of four categories: coupled, decoupling, coupling, or decoupled (Table 3). For the purposes of this study, we considered events in either the decoupling or coupling categories to be in a single “moving” category.

**Table 3.** Luke et al.’s four coupling status possibilities for a measured iMG event

	Post-impact status	
	On teeth	Off Teeth
*Pre-impact status*		
On teeth	Coupled	Moving (decoupling)
Off teeth	Moving (coupling)	Decoupled

Finally, our Salvaging technique includes a label for acceleration events based on their coupling status from our machine-learning classification algorithm, which detects mouthguard decoupling [24]. If the acceleration event is coupled, it is labeled highly reliable. If the acceleration event is decoupled, we use a wavelet salvaging process and re-check the coupling status with the classification algorithm. If the impact is converted to being labeled coupled, we consider it moderately reliable. Finally, if the impact is determined to be decoupled even after salvaging, we use a subset of the measurement duration (− 1 to 12 ms) in the salvaged signals to quantify peak kinematics [35]. This avoids deriving peak kinematics from late peaks, often the result of decoupling. We consider this final category of impact to have low reliability.

We compared labeling techniques by percent agreement and inter-rater reliability. We used a Cohen’s kappa to compare each labeling technique pairwise across the three available levels for each technique. We chose a pairwise comparison so that readers could determine differences between individual techniques. We also showed the effects of using each technique's “best” category label on the final distributions. We selected the superior category from each labeling technique and calculated the distribution statistics using only those measured events. To ensure a fair comparison across labeling techniques, we held the filtering constant at 200 Hz for linear and angular kinematics to make this comparison, using a 4th-order phaseless Butterworth filter [29]. A Kruskal–Wallis rank-sum test was used to report differences between distributions, followed by a post hoc pairwise Wilcoxon rank-sum test.

We used *p* = 0.05 as the *a priori* level of statistical significance for our tests. We completed all data post-processing in MATLAB R2023a (MathWorks–Natwick, MA) and used RStudio 2023.06.0 (R Foundation for Statistical Computing, Vienna, Austria) to calculate summary statistics and generate plots. The Virginia Tech Institutional Review Board (IRB) approved the field data collection under protocol 21-500.

## Results

### Post-Processing Technique Comparison

**Fig. 3.**
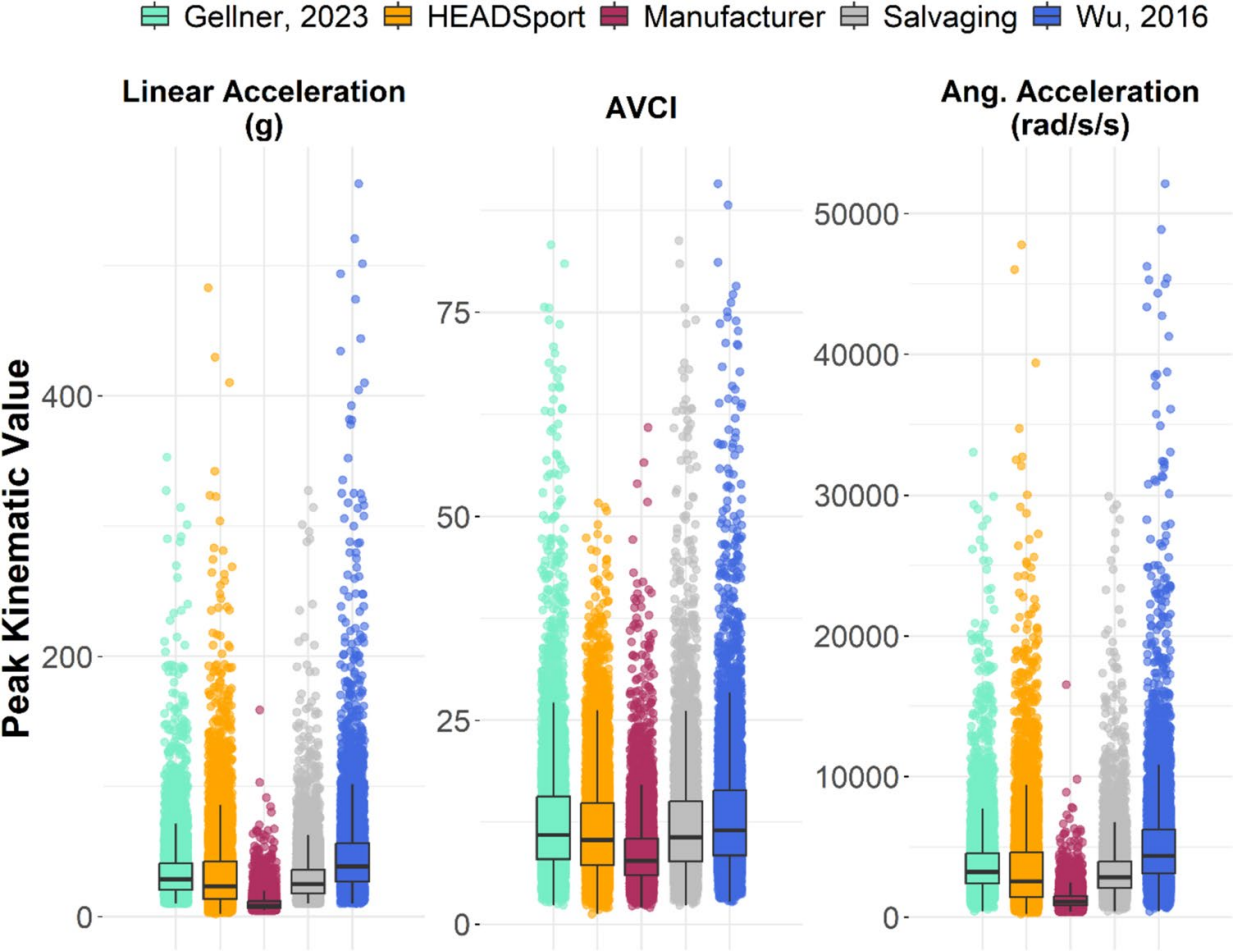
Overall distributions of iMG-measured acceleration events using different post-processing techniques. Noticeable differences in distribution characteristics, such as mean and scatter, can be observed between the tested post-processing techniques.

The different post-processing techniques changed the head acceleration event distribution statistics (Figure 3). These differences were larger with the distribution percentile, with the highest magnitude differences at the 95th percentile. All techniques except for the manufacturer’s processing technique had noticeable high-magnitude outliers in their distributions for each kinematic.

We ran a non-parametric Kruskal–Wallis test for rank differences and found significant differences between techniques. Post hoc pairwise comparisons found differences between all techniques for each of the three kinematic values. The magnitude of difference in mean, median, and 95th percentile varied by technique pair (Table 4). Our Salvaging algorithm, Gellner et al., and HEADSport were the most similar in mean values. Wu et al. and the manufacturer were most dissimilar, differing by 37.6 g in mean acceleration, 4.7 in mean AVCI, and 4050 rad/s^2^ in mean angular acceleration.

**Table 4.** Distribution statistics for the post-processed measured head acceleration events

Kinematic	Technique	Mean	SD	Percentile				
				5th	25th	50th	75th	95th
Peak linear acceleration (*g*)	Manufacturer	10.4	6.9	5.3	6.6	8.4	11.9	21.4
	Wu et al. [21]	48.0	38.0	16.3	26.8	38.7	56.7	105.0
	HEADSport	33.5	32.8	7.0	13.4	23.4	42.3	91.3
	Gellner et al. [29]	35.1	24.9	13.5	20.8	28.8	41.2	74.9
	Salvaging	30.3	21.1	12.1	18.1	25.1	35.9	63.4
Angular velocity change index	Manufacturer	8.9	4.7	4.2	6.0	7.8	10.5	16.8
	Wu et al. [21]	13.6	8.3	5.6	8.4	11.5	16.4	27.8
	HEADSport	11.9	6.5	4.6	7.3	10.3	14.8	24.5
	Gellner et al. [29]	13.0	7.9	5.3	8.0	10.9	15.7	26.1
	Salvaging	12.5	7.4	5.1	7.7	10.6	15.1	24.9
Angular acceleration (rad/s^2^)	Manufacturer	1252	692	545	837	1111	1482	2359
	Wu et al.[21]	5302	3935	1877	3121	4359	6213	11392
	HEADSport	3611	3439	787	1410	2559	4617	9755
	Gellner et al. [29]	3870	2587	1520	2398	3232	4528	8117
	Salvaging	3369	2211	1370	2100	2842	3970	6915

Differences increased with increasing percentiles. Methods had a maximum difference of 11 g at their 5th percentile but an 83.6 g maximum difference at the 95th percentile. Similarly, AVCI differed by 1.4 at the 5th percentile but 10.9 at the 95th, and angular acceleration had a 1332 rad/s^2^ difference at the 5th percentile but a 9032 rad/s^2^ range at the 95th percentile.

**Fig. 4.**
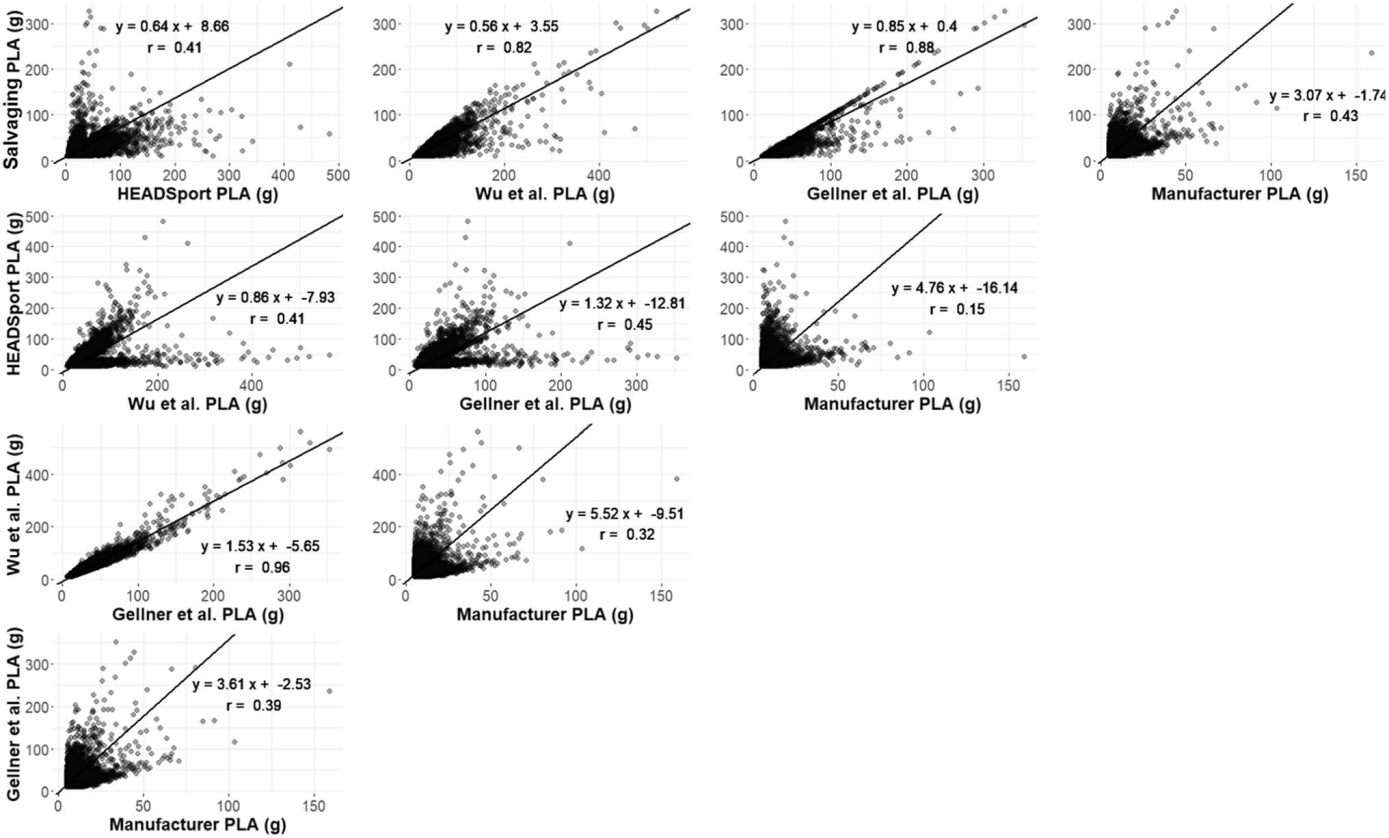
Scatter plots for each post-processing method against each other, with ordinary least products regression lines, equations, and Pearson correlation coefficient. Severe disagreement in high severity events were seen between the tested techniques. *PLA* peak linear acceleration.

Next, we used ordinary least products regression, which considers error to be present in both measures, to find the approximate scale factor between methods. Even methods with similar central distribution statistics show severe disagreement in many cases of peak kinematic values (Figure 4), which helps explain the differences in the upper tail of the distributions.

### Impact Labeling Comparison

We measured the agreement between labeling categories from the three types of impact labels: the manufacturer’s Quality metric, Luke et al.’s coupling status, and our Salvaging reliability flag (Figure 2). Percent agreement and inter-rater reliability were calculated for each labeling technique by category using their best, middle, and worst categories (Table 5). We found that the labeling techniques generally disagreed on a given impact and had low inter-rater reliability for a given category. Cohen’s Kappa was less than 0.07 for each pairwise rater comparison.

**Table 5.** Number of Accelerative Events Assigned to Each Category by the Three Labeling Methods

		Manufacturer Quality			Luke et al.*		
		0 (Best)	1	2 (Worst)	Coupled	Moving	Decoupled
Salvaging	High	**10%**	34%	17%	**10%**	32%	21%
	Med	4%	**15%**	14%	3%	**21%**	8%
	Low	0%	2%	**2%**	0%	2%	**1%**
Manufacturer Quality	0 (Best)				**2%**	5%	7%
	1				10%	**28%**	13%
	2 (Worst)				1%	22%	**9%**

Bold categories are matches between labeling methods

*Luke et al. percentages were calculated using only acceleration events that did not have an “unclear” coupling status

**Fig. 5.**
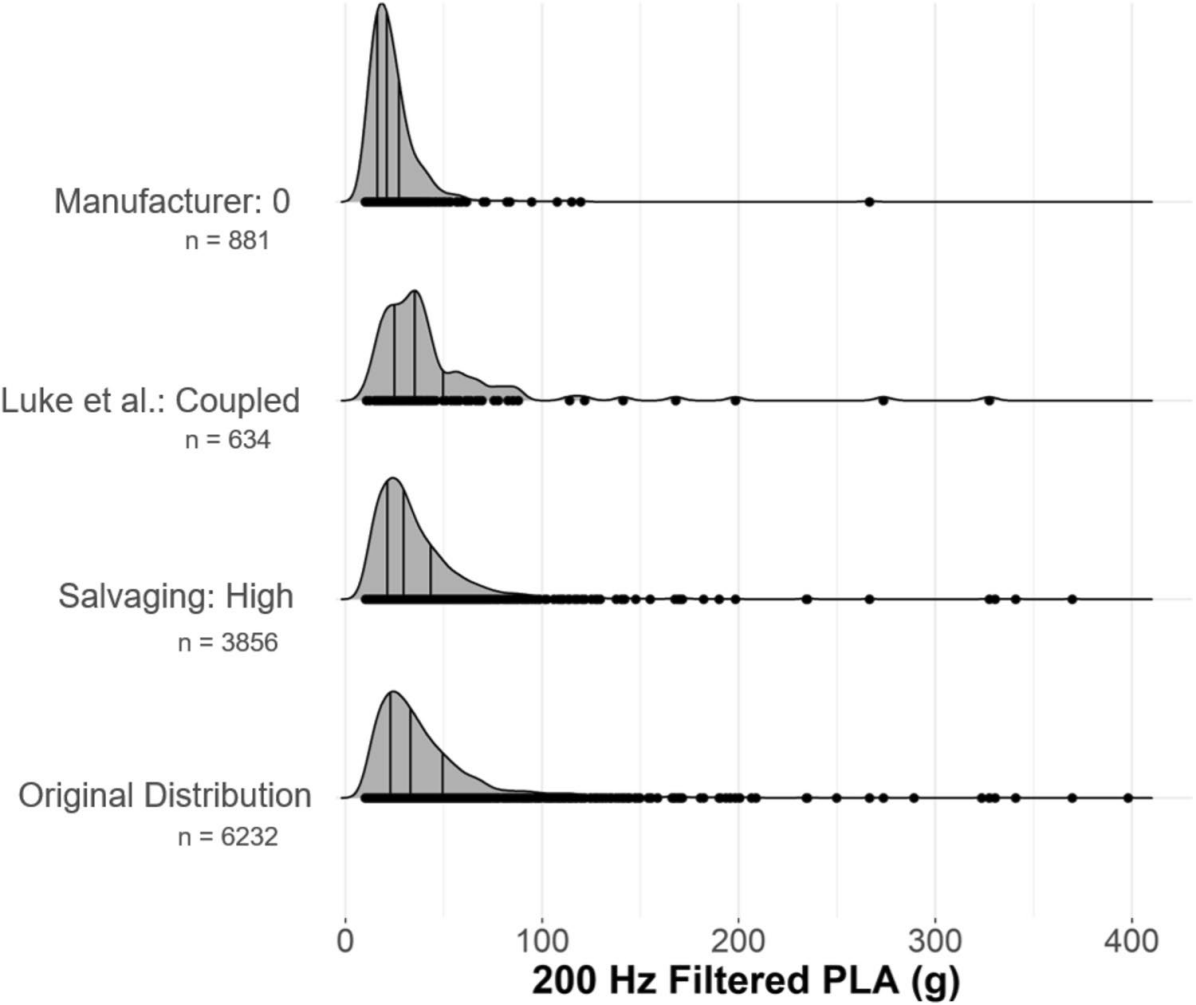
Peak linear acceleration distributions for all impacts in the highest quality category for each labeling technique. Distribution shapes and statistics differed from the original distribution and one another based on which labeling technique was used to select the “best” acceleration events to keep.

We also explored the effect of each labeling technique on a reported head acceleration exposure distribution. We used the labeling techniques to create three new distributions. Each distribution contained only acceleration events labeled as being the best by one of the techniques. For example, for the first distribution, we used only data that the manufacturer labeled as quality category “0.” Our comparison method resulted in three distributions, each with a different number of samples. We filtered all raw data using a 200 Hz cutoff frequency filter for both linear accelerometers and angular velocity sensors to make the quantitative comparison between distributions fair. In reality, the Salvaging technique could dictate a different effective filter for each impact, but we kept the filter cutoff frequency constant to avoid confounding factors when comparing distributions. We compared the resulting distributions to one another and to the original distribution with all acceleration events. The choice of labeling technique affected the final distribution’s mean, median, and 95th percentile peak linear accelerations (Figure 5).

## Discussion

Data post-processing was shown to alter head acceleration event kinematic magnitudes and distributions in our sample of female ice hockey field data. We tested five post-processing techniques, each shown to improve the accuracy of iMG signals in relation to laboratory instrumentation. These techniques differed from one another by as much as 391% in reported peak linear acceleration at the 95th percentile value of the distribution. Central statistics, such as the mean and median, had much higher agreement between the techniques.

Reported distributions will differ by post-processing technique. While central statistics may remain similar, large differences in the most severe head impacts may be problematic, as these impacts are generally the rare data points associated with injury. Quantifying a threshold for injury will be difficult when different post-processing techniques confound reported kinematics.

Some of the highest magnitude acceleration events in this dataset were likely unrealistic for the population of helmeted female ice hockey players from which we collected data [30, 36]. Due to study limitations, we could not video-verify every impact in this dataset, but we used the dataset as an example for comparisons between post-processing techniques. Therefore, this study can be viewed as a “worst case” for differences between post-processing and labeling techniques. As a confirmation, we did repeat the analysis on a subset of “best-case” head acceleration events that had been simultaneously labeled into the “best” category of each labeling technique (*n* = 80). Even with the best-case data, we found that peak kinematics could differ by up to 200%, although the trend was less pronounced for AVCI.

Unrealistically high data can be spotted rather easily by researchers, but unrealistically low data are much more difficult to find. Four of the five tested post-processing techniques agreed on the general distribution of peak kinematic values (Figure 3) and had reasonable OLP slope values for peak linear acceleration (0.56–1.53). The iMG manufacturer’s post-processed data were considerably lower than the other four techniques (OLP slopes: 3.07–5.52). This may indicate that the manufacturer’s technique generally underestimates event magnitudes in both linear and angular kinematics. Researchers should, therefore, expect data directly from the manufacturer of this iMG to have lower magnitudes than other post-processing techniques applied to iMGs.

Regression analysis verified that there was a range of correlations between the techniques. It is important to note that the Salvaging, HEADSport, and the manufacturer’s techniques all filtered each acceleration event differently based on signal characteristics. These three signal-specific post-processing techniques showed disagreement on outlier data points, which resulted in a bimodal-shaped plot with high-magnitude PLA in one category being low on the other axis and vice-versa. This is in contrast to the constant filtering applied by Gellner et al. and Wu et al. These inherent differences influenced the ability of each technique to correlate with one another in our regression analysis.

Labeling specific head acceleration events as high quality also influenced the final reported distributions. Researchers should report their labeling method and data proportions in each category when reporting head acceleration distributions from these devices. We should also be aware of this potential effect when reviewing literature, especially when specific categories of data are favored or processed differently than others and then lumped into one distribution. In this dataset, removing lower-quality categories had a large effect on distributions. The distributions, their median, 95th percentile, and means differed based on which labeling method was used to select the “best” quality acceleration events. Whatever the labeling technique, researchers should consider reporting distributions with and without their lower-quality categories to quantify the effects of the labeling. Another alternative may be to apply multiple labeling techniques, selecting only the best in each. This would likely lead to higher-quality signals in the final dataset but would certainly reduce the sample size of available head acceleration events for analysis.

A field-relevant iMG data post-processing method recommendation would greatly benefit the field of head impact biomechanics. Thus, we propose that the field moves toward two standard, validated post-processing techniques: first, post-process all kinematic signals using a laboratory-validated iMG filtering technique that most effectively reduces peak signal error under idealized conditions [18]; second, find and deal with specific signal artifacts that deviate from idealized conditions using an advanced technique such as HEADSport [19]. In all cases, we recommend that the techniques be specified and the proportion of data in each possible filtering category be clearly stated. This more commonized post-processing framework will create greater transparency and comparability across studies and user populations where iMGs are applied.

This study has several limitations. We were not able to video-validate all head acceleration events, meaning some of the events in this dataset may not represent relevant events. Even still, these outlier signals were included because they can be present in real-world datasets. Depending on the efficacy of the video review process, unrealistic events may leak into final head acceleration event distributions. These high-magnitude events also helped to characterize a worst case for our comparison of methods. Therefore, we believe it is relevant to test with these edge cases. Next, when comparing labeling techniques, the manufacturer’s Quality label may not be meant to detect decoupling; Luke et al.’s method and the Salvaging method are meant to detect and label decoupling specifically. The manufacturer label may be meant to catch other data artifacts in addition to or instead of decoupling, which could be one reason why we saw disagreement between this method and the others. Additionally, we chose to filter all data with a 200 Hz cutoff frequency filter to compare the labeling effects on the final distributions. The Salvaging technique would have used the optimal filters based on Gellner et al. for high-quality data. This would have influenced the final distribution magnitude statistics but not the number or selection of data points in the final distribution. Finally, our data come from a single population (female ice hockey players) in a helmeted sport; therefore, these suggestions are valid for this and similar populations, but should be confirmed for other populations, such as an unhelmeted male sport.

## Conclusions

Instrumented mouthguards offer the opportunity to study volunteer athlete populations’ head acceleration exposure. However, post-processing and signal labeling can influence the magnitude of the reported exposure. Advanced signal processing techniques such as HEADSport or our salvaging algorithm should be used alongside video review [37] and well-studied labeling techniques [24] to ensure the best-quality data are included and appropriately post-processed on a signal-by-signal basis when reporting head acceleration exposure data. Commonizing methods across studies will enable better cross-study comparisons.
